# Dependence of Acute Myeloid Leukemia on Adhesion within the Bone Marrow Microenvironment

**DOI:** 10.1100/2012/856467

**Published:** 2012-01-04

**Authors:** Pamela S. Becker

**Affiliations:** Division of Hematology, Institute for Stem Cell and Regenerative Medicine, University of Washington, Campus Box 358056, 815 Mercer Street N415, Seattle, WA 98109, USA

**Keywords:** cell adhesion, drug resistance, CXCR4 receptor, integrin alpha4beta1, tumor microenvironment.

## Abstract

Acute myeloid leukemia (AML) cells home to the endosteal region of the bone marrow. They interact with bone marrow stromal components including extracellular matrix proteins, glycosaminoglycans, and stromal cells, by which they derive proliferative and growth inhibitory signals. Furthermore, adhesion to marrow stroma confers chemotherapy drug resistance and thereby promotes leukemia survival. A subpopulation of the leukemic blasts, known as leukemia stem cells, that are capable of propagating the leukemia, remain sheltered in the bone marrow microenvironment, exhibit resistance to chemotherapy, and serve as the origin of relapse after a variable period of remission. Detachment of these cells from the bone marrow in combination with chemotherapy may improve the outcome of therapy for AML.

## 1. Adhesion and Leukemia Biology

**Figure 1:**
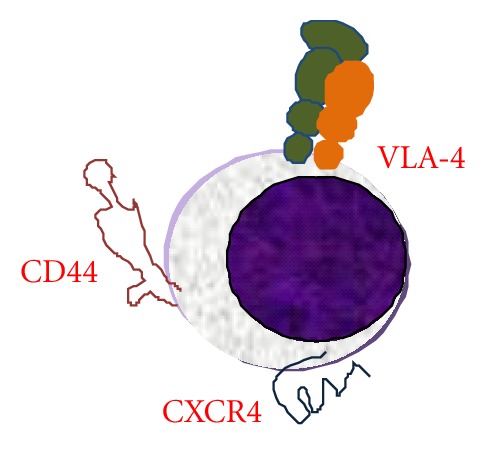
Leukemia cell adhesion and chemokine receptors in stromal interactions. CXCR4, VLA-4, and CD44 play critical roles in leukemia cell homing and migration.

Adhesive properties of leukemia cells are likely responsible for the complication of leukostasis in AML as well as leukemic meningitis, leukemia cutis, extramedullary leukemia, and formation of chloromas. Three receptors, VLA- (very late antigen-) 4, CXCR4, and CD44, play a critical role in normal stem cell homing and also appear to be paramount to the homing of AML cells to, or retention within, the bone marrow. VLA4 is the *α*_4_ *β*_1_ integrin that mediates adhesion to alternatively spliced fibronectin and cellular vascular cell adhesion molecule-1 (VCAM1). CXCR4 is a chemokine receptor for stromal derived factor-1 (SDF-1) also known as CXCL12. CD44 is a hyaluronic acid receptor that is an E selectin ligand expressed by hematopoietic stem cells known as HCELL when properly glycosylated [1]. The VLA-4/VCAM-1 pathway has been implicated in the attachment of leukemic blasts to the vessel wall [2]. Both CXCR-4 and VLA-4 mediate migration of AML blasts [3, 4]. The role of CXCR4 in leukemia retention was illustrated by experiments that demonstrated reduction of primary human AML cell numbers previously engrafted in immunodeficient NODscid mice with antibody to CXCR-4 [5]. In contrast, there was no decrease in normal human CD34+ cell numbers in mice engrafted with normal human cord blood mononuclear cells after treatment with the anti-CXCR4 antibody [5]. This finding highlights the exquisite ongoing dependence of the engraftment of human AML in immunodeficient mice on CXCR-4. In addition, the CD44 hyaluronic acid receptor is involved in homing of normal human CD34+ cells [6]. Similarly, administration of an antibody to CD44 blocked engraftment of AML cells in NOD-scid mice [7]. Furthermore, high level expression of CD44 by leukemia cells was sufficient to generate leukemia by leukemia-initiating cells even after withdrawal of overexpression of the HoxA10 gene that initiated the leukemia [8]. Thus, at least three adhesion mechanisms, CXCR4/SDF1 (CXCL12), VLA-4/VCAM-1 or fibronectin, and CD44/ligand, function in acute myeloid leukemia migration, retention, and survival (Figure 1). 

Not only are leukemia cells dependent on the bone marrow stroma for survival, but also they are capable of distorting normal bone marrow niches in a manner that affects normal hematopoietic progenitor cells [9]. The endosteal region was the location identified as the site of homing of chemotherapy-resistant AML stem cells [10], supporting the concept of localizing niches for certain cell types, and this location is the same region as a niche for normal homing hematopoietic stem cells [11].

## 2. Adhesion and Chemotherapy Resistance

 Adhesion of acute myeloid leukemia cells confers resistance to several chemotherapy agents, including cytarabine, one of the most active agents in AML. This ability is known as environment-mediated drug resistance (EMDR) [12]. The theory is that this capacity, particularly when possessed by leukemia stem cells, for example, gives rise to minimal residual disease, which in turn, is the origin of relapse after a period of genetic instability and acquisition of more complex drug resistance. Growth of AML cells on HS-5 stroma reduced daunorubicin- or cytarabine-induced apoptosis [13]. Adhesion of U937 to fibronectin via *β*_1_ integrins inhibits mitoxantrone- and etoposide-induced apoptosis [14]; similarly, adhesion of U937 or HL60 leukemia cell lines to fibronectin inhibited daunorubicin or cytarabine induced apoptosis [15]. Adhesion of primary patient AML cells to fibronectin or immobilized VCAM-1 conferred resistance to cytarabine or daunorubcin plus cytarabine [16]. Agents that block adhesion mediated by VLA-4, including a fibronectin peptide [17], antibody to VLA-4 [15, 16], soluble VCAM-1 [16], a small molecule inhibitor of VLA-4 [18], or a peptide inhibitor of CXCR-4 [19] all overcame adhesion mediated chemotherapy resistance. Moreover, a peptide inhibitor of the chemokine receptor, CXCR4, exhibited direct cytotoxicity against AML and multiple myeloma cells in vitro and in xenografts [20]. The CXCR4 inhibitor AMD3100 worked synergistically with histone deacetylase inhibitor panobinostat to induce apoptosis of AML cells in vitro [21]. Another CXCR4 inhibitor, AMD3465, interfered with chemotaxis of AML cells toward SDF1 in vitro, prevented SDF1-induced activation of survival pathways in AML cells, caused mobilization of human leukemia cells in immunodeficient mouse xenografts, and enhanced the activity of sorafenib in Flt3-positive AML [22]. In an in vivo murine model of acute promyelocytic leukemia, AMD3100 mobilized leukemia cells into the blood and, in combination with cytarabine, reduced leukemic burden and prolonged animal survival [23]. These latter two studies demonstrated the proof of principle that the concept that a combination of a CXCR4 inhibitor with chemotherapy or targeted therapy was efficacious in enhancing leukemia cytotoxicity in vivo. 

Several potential mechanisms have been proposed for the ability of integrin-mediated signaling to protect from chemotherapy toxicity that involve activation of survival pathways or inhibition of apoptosis. The specific pathways include activation of the PI3 K/Akt/bcl-2 pathway [15], an interaction between Wnt and adhesion-dependent signaling pathways [24], and increased degradation of proapoptotic bcl-2 family member Bim [25]. Integrin-linked kinase (ILK) also plays a role in the activation of Akt upon adhesion of AML cells [26, 27].

## 3. Chemokine Receptor or Adhesion Receptor Expression and Prognosis

 Expression of each of the two receptors, VLA-4 or CXCR4, has been associated with prognosis in AML; the former is correlated with better survival, and the latter portends worse survival. Several studies showed that high-level expression of CXCR4 was associated with poor prognosis in AML. As described earlier, CXCR-4 was demonstrated to have a pivotal role in the homing, migration, and development of human AML in the NODscid murine mutant [5]. Although not all AML patient cells tested exhibited surface expression of CXCR-4 with average expression 24%, all AML cells analyzed uniformly exhibited internal expression of CXCR-4 after permeabilization and labeling [5]. AML patients with high-level (≥20%) expression of CXCR-4 by the CD34+ population exhibited reduced overall survival and relapse free survival [28]. By multivariate Cox regression analysis, high CXCR-4 expression had a relative risk for relapse of 13.4 (*P* < 0.001) [28]. Furthermore, an independent study also corroborated that high-level CXCR-4 expression predicted overall and event-free survival in patients with normal karyotype and unmutated Flt3 status [29], and lower expression of CXCR4 correlated with longer relapse-free and overall survival [30] or higher complete remission rate [31]. Presence of functional circulating CXCR4 bearing microparticles was correlated with high white blood count in AML patients and was proposed to be involved in AML progression, possibly by promoting dissemination of leukemia [32]. 

 In contrast to CXCR4 expression, high-level VLA-4 expression has the opposite effect on prognosis in AML. Higher functional expression of VLA-4 was shown to correlate with longer survival for newly diagnosed adult AML [16]. Furthermore, higher expression of VLA-4 by flow cytometry correlated with better prognosis of pediatric AML patients [33]. These two large studies are in contrast with an earlier, smaller trial that suggested that VLA-4 expression conferred poor prognosis [15]. The precise mechanism for this improved survival is unknown, but one hypothesis is that as soluble VCAM-1 (sVCAM-1) levels are elevated in AML [34], the AML blasts may be dislodged from the bone marrow due to binding of sVCAM-1 and thus be more susceptible to chemotherapy.

## 4. Clinical Trials of Adhesion Inhibitors in AML

There are several ongoing clinical trials utilizing this novel concept of combining agents that mobilize leukemia with chemotherapy (Table 1). For example, there is an ongoing multicenter phase I trial of plerixafor in combination with standard induction “7 + 3” chemotherapy in AML including high-dose daunorubicin 90 mg/m^2^ daily for three days. There is also an ongoing multicenter phase I trial of an anti-CXCR-4 antibody in combination with mitoxantrone, etoposide, and cytarabine for relapsed/refractory AML. As these inhibitors enter the clinic, we will ascertain their ability to mobilize AML out of the protected marrow microenvironment and determine if this approach improves outcome of patients with new diagnosis or relapsed/refractory AML.

## 5. Future Prospects

 In summary, there may be several critical mechanisms for adhesion of AML within the bone marrow, and discovery of novel mechanisms and novel inhibitors targeting disruption of adhesion may provide a significant advance in the treatment of AML. 

## Figures and Tables

**Table 1:** Clinical trials of combinations of adhesion inhibitors and chemotherapy for AML.

Title	Clinicaltrials.gov designation	Institution or sponsor

Study of plerixafor combined with cytarabine and daunorubicin in patients with newly diagnosed acute myeloid leukemia	NCT00990054	Multicenter-Genzyme-Sanofi

First in human study to determine the safety, tolerability, and preliminary effectiveness of MDX-1338 (BMS936564) in subjects with acute myelogenous leukemia (AML)	NCT01120457	Multicenter-Bristol-Myers Squibb

Granulocyte colony-stimulating factor (G-CSF) and plerixafor plus sorafenib for acute myelogenous leukemia (AML) with FLT3 mutations	NCT00943943	MD Anderson Cancer Center

Chemosensitization with plerixafor plus G-CSF in acute myeloid leukemia	NCT00906945	Washington University

IV plerixafor with mitoxantrone etoposide and cytarabine for acute myeloid leukemia (AML)	NCT01027923	Washington University

Plerixafor and clofarabine in frontline treatment of elderly patients with acute myelogenous leukemia (AML)	NCT01160354	MD Anderson Cancer Center
